# Presynaptic Terminal Proteins and Nicotinic Receptors Are Depleted from Mouse Parasympathetic Ganglionic Junctions Paralysed with Botulinum Neurotoxin Type A †

**DOI:** 10.3390/toxins18010043

**Published:** 2026-01-14

**Authors:** Ahmed Al-Sabi, Gary W. Lawrence

**Affiliations:** 1 School of Biotechnology, Dublin City University, D09 K20V Dublin, Ireland; 2 College of Integrative Studies, Abdullah Al Salem University, Khaldiya 72303, Kuwait

**Keywords:** acetylcholine, botulinum neurotoxin, ganglionic neurotransmission, hypersalivation, salivary duct, submandibular ganglion, synaptic plasticity, SNAREs

## Abstract

Plasticity is fundamental to the development, strengthening, and maintenance of healthy synaptic connections and recovery from injury in both the central and peripheral nervous systems. Yet, the processes involved are poorly understood. Herein, using a combination of patch-clamp electrophysiology and immuno-fluorescence confocal microscopy in adult mice, it is shown that blockade of synaptic transmission at submandibular ganglion junctions exposed to botulinum neurotoxin type A was accompanied by a rapid and striking decline in the abundance of synaptic vesicle markers—SV2, vesicle-associated membrane protein 2, and vesicular acetylcholine transporter—plus SNAP-25 (cleaved and intact) and postsynaptic α7 nicotinic acetylcholine receptors. Such alterations by the neurotoxin of parasympathetic synapses contrast starkly with the stability of postsynaptic proteins at nearby skeletal neuromuscular junctions. Both neurotransmission and the expression of SV2 and α7 nicotinic acetylcholine receptors remained depressed for 4 weeks, with full recovery of synaptic function delayed for more than 8 weeks. These novel findings may explain the relatively slow recovery of autonomic function after botulism or following therapeutic injections to alleviate hypersecretory disorders.

## 1. Introduction

The neuromuscular junctions (NMJs) in adult mice are resolute, retaining recognisably similar structures for months and only reverting to plastic growth in response to injury or toxic insults that cause neuroparalysis [1,2,3]. By contrast, healthy parasympathetic synaptic connections in the murine submandibular ganglion (SMG) remain highly dynamic well into adulthood, continuously retracting presynaptic fibres and growing new synapses [4,5]. Growth of motor nerve terminal sprouts expedites the recovery of NMJs paralysed with botulinum neurotoxin type A (BoNT/A) [2], a protease that inhibits presynaptic neurotransmitter release by cleaving synaptosomal-associated protein of Mr = 25k (SNAP-25) [6]. Recovery from neuromuscular inhibition by BoNT/A also involves compensatory plastic changes in muscle resting membrane potential, quantal size, and voltage-gated calcium channels [7]. There is intense interest in the role of neuronal plasticity in NMJ recovery because of the widespread clinical use of BoNT/A as a long-duration (3–6 months) [8] muscle relaxant (e.g., for dystonia, spasticity) [9]. By contrast, little is known regarding the effect of long-term paralysis on the parasympathetic nervous system. Such investigations are warranted because they could elucidate a basis for the longer duration of clinical benefit achieved for some patients when treated for autonomic disorders (e.g., for hyperhidrosis, sialorrhea; 3–12 months) after a single treatment with BoNT/A [10]. Herein, we hypothesize that prolonged paralysis by BoNT/A of cholinergic neurotransmission in parasympathetic SMG might involve the perturbation of pre- and postsynaptic machinery involved in the dynamic plastic activity described above, a process distinct from the stability observed at the NMJ.

BoNT/A was applied unilaterally to the salivary ducts in mice, and the extent and duration of inhibition of parasympathetic neurotransmission at the SMG were assessed by electrophysiological recording. In conjunction, changes in the abundance of biochemical markers were revealed by immunofluorescence microscopy and Western blotting. Strikingly, inhibition of synaptic transmission caused a severe decline in ganglia of presynaptic markers chosen for their relevance to cholinergic neurotransmission and its inhibition by BoNT/A [8] (synaptic vesicle protein 2 (SV2), which is a constituent of the high-affinity receptors exploited by BoNT/A to enter nerve terminals; vesicle-associated membrane protein 2 (VAMP2 or synaptobrevin 2), which binds with SNAP-25 and syntaxin 1 to mediate the synaptic vesicle fusion that enables neurotransmitter exocytosis, and is a substrate for other BoNT serotypes; vesicular acetylcholine transporter (vAChT), which fills synaptic vesicles with the neurotransmitter acetylcholine) and postsynaptic α−bungarotoxin (α−BuTX) sensitive α7 sub-type nicotinic acetylcholine receptors (α7nAChRs), which contribute to the reception of acetylcholine transmission by ganglionic neurons [11]. It is concluded that neurotransmission is critical for the maintenance of presynaptic terminal proteins and clusters of postsynaptic α7nAChRs in the mouse SMG.

## 2. Results

### 2.1. Potent and Persistent Blockade of Synaptic Transmission in SMG from Mice Injected with BoNT/A

**Figure 1:**
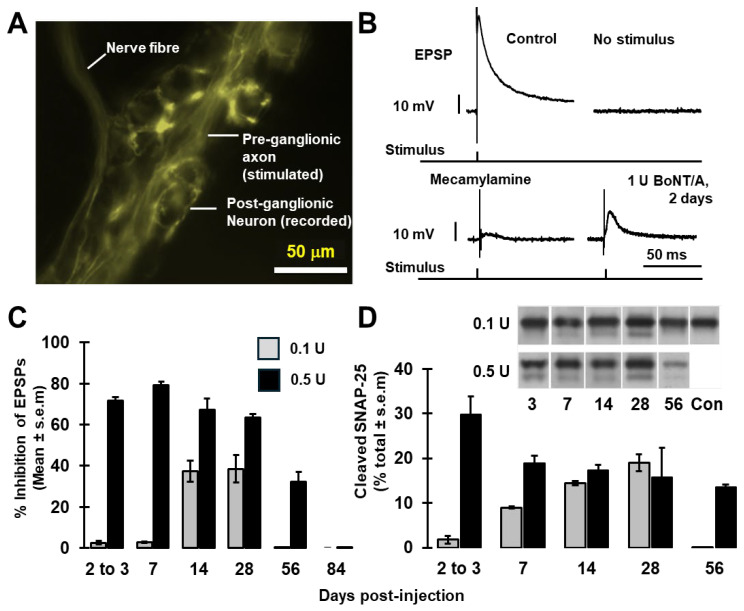
Pertinacious suppression by botulinum neurotoxin type A (BoNT/A) of cholinergic transmission in the mouse submandibular ganglion (SMG). (**A**) Yellow fluorescent protein (YFP) expressing lingual nerve fibres innervating SMG somata. (**B**) Excitatory postsynaptic potentials (EPSPs) elicited by stimulating the lingual nerve were almost abolished by 0.2 mM mecamylamine and greatly reduced 2 days after the topical application of 1 U BoNT/A to SMGs in vivo. (**C**) Prolonged reduction in EPSP amplitudes by 0.1 U (grey bars) and 0.5 U (black bars) of BoNT/A. Mean values from four to nine cells per point are plotted (taken from two to four mice). (**D**) Western blotting confirmed the persistence of BoNT/A protease throughout the period of inhibition; note that samples contain both preganglionic nerve fibres and postganglionic nerve cells plus fibres. Con: control sample from an untreated mouse. The graph displays quantification of synaptosomal protein of Mr = 25 k (SNAP-25) cleavage from Western blots of samples from one animal (0.1 median lethality units [U], days 3, 7, 14, and 28; 0.5 U day 14) and mean values from two mice (0.1 U, day 56; 0.5 U, days 7, 28, and 56) or three mice (0.5 U, day 3).

In SMG from mice that have been genetically modified to express yellow fluorescent protein (YFP) under the control of the Thy1 cell surface antigen (THY1) promoter (Thy1 YFP mice [12]), many of the pre-ganglionic axons innervating submandibular neurons strongly express YFP, facilitating their visualisation and indirect localisation of postsynaptic somata of ganglionic neurons for electrophysiological recording (Figure 1A).

In control mice, excitatory postsynaptic potentials (EPSPs) with amplitudes of 58 ± 1 mV (*n* = 12 cells from five animals) were evoked in the latter neurons after stimulating the sublingual nerve (Figure 1B); spontaneous EPSPs were not observed, in accord with a previous report [13]. The EPSPs were suppressed by application of mecamylamine, a non-competitive, non-selective nAChR antagonist (Figure 1B), confirming that they are elicited by acetylcholine-mediated neurotransmission. Accordingly, it was found that prior injection of BoNT/A into mice 2 days before recordings depressed the EPSP amplitudes (Figure 1B). The level of inhibition increased from ~3% after injection of 0.1 U BoNT/A to ~80% following 1 U (Figure 1B,C).

For studying the persistence of the blocking activity of BoNT/A, two doses (0.1 and 0.5 U) were administered because doses of 1 U or higher induced signs of respiratory distress by 24–48 h in some cases. Mice were kept for up to 12 weeks prior to functional and biochemical evaluation. At 0.1 U BoNT/A, EPSP amplitudes showed a delayed and considerable inhibition (40 ± 7%, *n* = 4) that persisted for up to at least 28 days (Figure 1C) but recovered by 56 days post-treatment.

On the other hand, a higher dose (0.5 U) reduced EPSP amplitudes (~70%) within 3 days, reaching a minimum at day 7 before gradually recuperating. Note that neurotransmission remained significantly suppressed for at least 8 weeks, but with recovery by 84 days. Western blotting using antibodies specific for the N-terminal of SNAP-25 showed that the higher dose induced a faster onset of its cleavage and a longer persistence of the protease activity (Figure 1D and Figure S1), which corroborates the functional recordings. Only a small minority of the SNAP-25 was proteolysed, which likely reflects a preferential cleavage of SNAP-25 at presynaptic sites of neurotransmitter release; this accords with previous findings at NMJs [3,14] and for blockade of cholinergic transmission between rat sympathetic superior cervical ganglion neurons in vitro [15]. Collectively, these data indicate that murine SMG cholinergic synapses are highly susceptible to BoNT/A treatment in vivo and that inhibition persists for >2 months.

### 2.2. Labelling in SMGs of SNAP-25 and Synaptic Vesicle Markers, SV2 and VAMP2, Is Decreased upon Blockade of Neurotransmission

**Figure 2:**
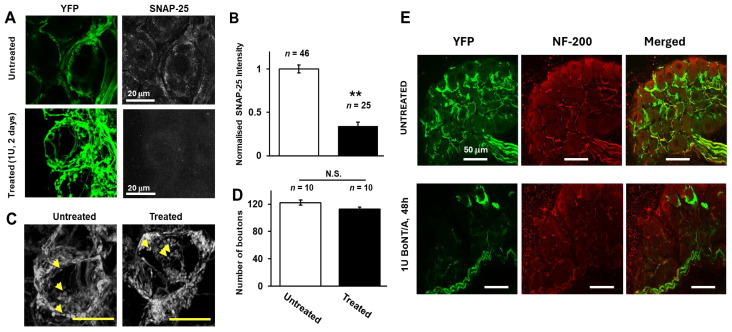
Inhibition of neurotransmission in mouse SMG is accompanied by the disappearance of SNAP-25 without changes in neurofilament 200 (NF-200). (**A**) Confocal z-stack micrographs of SMG neurons treated with or without 1 U BoNT/A showing the YFP-positive presynaptic axons plus boutons and SNAP-25 stained with monoclonal SMI 81. (**B**) Treatment resulted in a significant reduction in SNAP-25 (** *p* < 0.01; N.S., not significant; *n* values represent the number of microscope images analysed, taken from four mice). (**C**,**D**) Counting of YFP-labelled boutons revealed no significant difference between treated and non-treated SMG neurons (*p* = 0.089; *n* = 10 from four mice for both conditions). Arrowheads indicate YFP-labelled boutons in treated and non-treated SMGs. (**E**) BoNT/A did not cause any discernible effect on immuno-labelling for NF-200, which is visible in a subset only of YFP-expressing nerve fibres from ganglia of three mice.

Immunostaining and confocal analysis revealed that blocking neurotransmission caused a reduction in signal intensities for SNAP-25 in SMG treated with 1 U BoNT/A for 2 days (Figure 2A, lower panel) compared to untreated tissue (Figure 2A, upper panel). Quantification showed that this decrease is highly significant (Figure 2B; *p* < 0.01). This finding accords with a previous report of a decline in SNAP-25, in addition to its cleavage, in the submandibular gland of rats following the injection of BoNT/A into the gland [16]. Not all preganglionic nerve fibres exhibit equal expression of YFP [12], and it was observed herein that the proportions of ganglionic neurons innervated by bright YFP-expressing presynaptic fibres varied between animals and between individual ganglia in the same animal. Therefore, it was not possible to attribute differences in the number of neurons innervated by YFP fluorescent fibres to treatment with BoNT/A. However, there was no change in the number of fluorescent boutons associated with cells that were innervated with YFP-expressing fibres [untreated vs. 1 U BoNT/A: 122 ± 4 (*n* = 10) and 113 ± 3 (*n* = 10), respectively, *p* = 0.089, Figure 2C,D]. The staining observed for NF-200 (a marker for myelinated nerves that is not expressed in boutons) in mice treated with 1 U BoNT/A for 48 h (Figure 2E) appeared undiminished compared to control SMGs, indicating that neuroparalysis did not affect this axonal protein.

**Figure 3:**
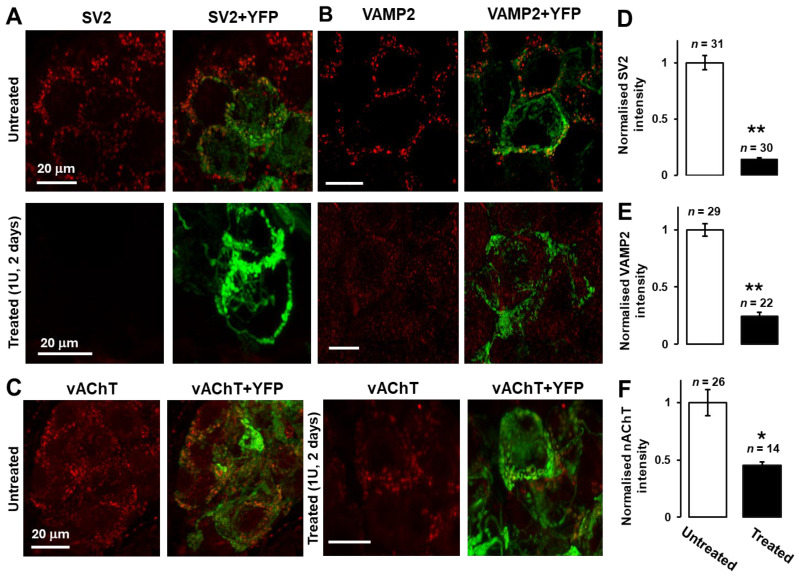
Presynaptic vesicle markers are down-regulated after SMG paralysis. Punctate immuno-labelling of synaptic vesicle protein (SV2) (**A**), vesicle-associated membrane protein 2 (VAMP2) (**B**), or vesicular acetylcholine transporter (vAChT) (**C**) in peri-somatic fibres around SMG neurons was greatly reduced by BoNT/A. (**D**) SV2 (** *p* < 0.01), (**E**) VAMP2 (** *p* < 0.01), and (**F**) vAChT (* *p* < 0.05). The *n*-values presented above the bars represent micrographs acquired from four mice for each of the proteins analysed.

SV2 was chosen as a classical presynaptic marker known to be expressed in SMG at different ages [5]; also, this protein acts as an acceptor for BoNT/A [17]. Accordingly, SV2 showed strong peri-somatic signals consistent with it being concentrated at the boutons, but not on the presynaptic axons or postsynaptic somata in SMG from control mice (Figure 3A, upper panel). Strikingly, SV2 was diminished significantly (*p* < 0.01, Figure 3D) in the BoNT/A-treated SMG neurons compared to the control. Likewise, immuno-labelling revealed similarly significant declines in the presynaptic vesicle proteins VAMP2 (Figure 3B) and vAChT (Figure 3C) (*p* < 0.01 and *p* < 0.05, respectively; Figure 3E,F) following inhibition of neurotransmission. Notably, the residual VAMP2 and vAChT were not concentrated at the YFP-stained terminals, though they still presented punctate patterns.

### 2.3. Silencing of Neurotransmission Depletes α7-nAChR on Postsynaptic SMG Neurons; Both Pre- and Postsynaptic Markers Remain Depressed in SMG for Several Weeks

**Figure 4:**
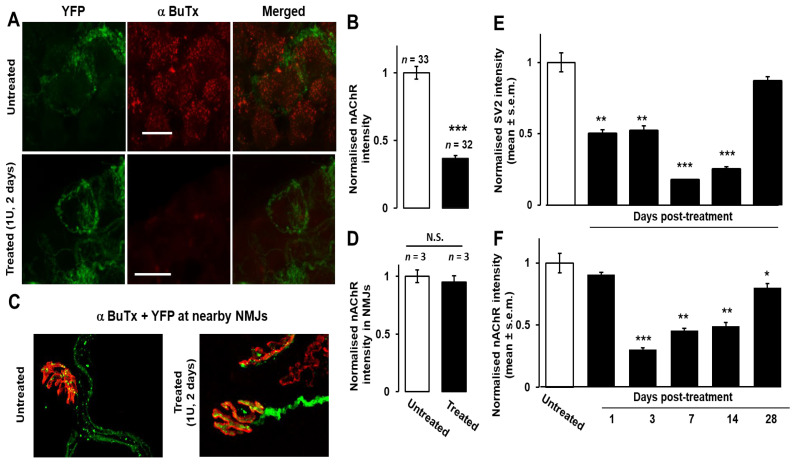
BoNT/A diminished postsynaptic α7 nicotinic acetylcholine receptors (α7nAChRs) in SMGs, but not nearby neuromuscular junctions (NMJs); SV2 and α7nAChR levels remained depressed for 28 days in SMGs. (**A**,**B**) In contrast to YFP, the α7nAChRs had virtually disappeared (***; *p* < 0.001) from SMGs of mice injected 48 h previously with 1 U BoNT/A, as determined from a lack of labelling with α-BuTX-fluor. (**C**,**D**) At nearby NMJs, α-BuTX-fluor binding was not reduced (N.S., not significant). (**E**) SV2 significantly reduced within 1 day (**; *p* < 0.01, ***; *p* < 0.001) of 0.5 U BoNT/A application. The signal intensities for SV2 (**E**) and α7nAChRs (**F**) remained significantly lower than in untreated specimens for at least 28 days. The *p*-values for SV2 and nAChR at day 28 are 0.067 (*n* = 20) and 0.016 (*n* = 10), respectively (*; *p* < 0.05, **; *p* < 0.01, ***; *p* < 0.001). The *n*-values shown in (**B**,**D**) represent the number of micrographs obtained from ganglia of three to four animals. The *n* values for (**E**) are 11–20 SMG neurons, and for (**F**) are 10–11 SMG neurons, imaged from the ganglia of three to four mice.

As reported by [11], clusters of postsynaptic nAChRs in SMG were detected using fluor-tagged α−BuTX, accompanied by a lower intensity of more diffuse labelling attributable to non-clustered receptors (Figure 4A, upper panel), indicating abundant α7nAChRs that could also be detected by Western blotting of solubilised extracts from the salivary ducts (Figure S2). Strikingly, staining with α−BuTX was virtually abolished in 1 U BoNT/A-paralysed SMGs (Figure 4A, lower panel, Figure 4B, *p* < 0.001), indicating a near-complete and extremely significant loss of α7nAChR binding sites compared to untreated tissue. In stark contrast, no decrease was visible in the labelling of nAChRs in nearby NMJs in the digastric muscle from mice pre-treated with 1 U BoNT/A for 48 h (Figure 4C,D). Thus, the down-regulation of post- and presynaptic proteins induced by inhibition of neurotransmission with BoNT/A is a phenomenon specific to the ganglionic synapse. In the latter, significant reductions in SV2 and α7nAChR signals were detectable from day 1 and 3 post-treatment, respectively, and persisted for several weeks, remaining significantly lower until day 28 after administration of 0.5 U BoNT/A, although partial recovery was evident by this time (Figure 4E,F). By contrast, the expression of SV2 and nAChRs was unchanged in the SMG of mouse tissue treated with an atoxic form of BoNT/A with an inactivated, mutated protease (BoTIM/A, [18]; see Figure S3). Thus, the compounded effect of BoNT/A on post- and presynaptic markers correlates with the long-lasting inhibition and eventual recovery of cholinergic neurotransmission.

## 3. Discussion

Presynaptic blockade in vivo by BoNT/A of neurotransmission in murine SMGs was accompanied by a striking decline in presynaptic SV2, vAChT, and VAMP2 and postsynaptic α-BuTX-binding sites that are likely to be α7 subunits of nAChRs. The expression of SNAP-25 in the SMG was also reduced, which accords with a previous report that SNAP-25 expression levels were reduced in the rat submandibular gland after the injection of BoNT/A [16]. Presumably, such losses contribute to the protracted inhibition of parasympathetic neurotransmission, in addition to the proteolytic inactivation of presynaptic SNAP-25. Indeed, the recovery of full-length SNAP-25 to pre-treatment expression levels correlated with the recovery of saliva secretion in rats after the injection of BoNT/A into their submandibular gland [16]. Although only some pre-ganglionic fibres brightly express YFP, it was detected in pre-terminal axons and presumed presynaptic terminals where it co-localised with the immuno-staining for SV2, vAChT, or VAMP2 [5]. Nevertheless, for cells that were innervated with YFP-expressing fibres, the number of fluorescent boutons per cell was undiminished in BoNT/A-intoxicated ganglia. Likewise, axonal NF200 immuno-reactive signals appeared unaffected by BoNT/A treatment. Thus, it is deduced that neuroparalysis of SMGs induced within days a selective loss of presynaptic proteins and postsynaptic α7nAChR, without an overall reduction in the numbers of pre-terminal axonal fibres and peri-somatic boutons within the ganglion. The depletion of a ganglionic nAChR was unexpected, given the hypersensitisation of salivary glands to acetylcholine (and other transmitters) after BoNT/A intoxication, but it is not incompatible with the latter because hypersensitisation is a glandular response to hypo-stimulation of muscarinic acetylcholine receptors [19,20].

The disappearance of synaptic markers in the intoxicated SMG is very different from the stability observed at nearby NMJs, which accords with previous findings that motor endplates in BoNT/A-paralysed sternomastoid muscle retain recognisable structures for many weeks until full functional recovery has occurred [2]. Unlike SMG synapses, paralysed motor endplates grow neurites that eventually form small extra-junctional synapses; these are only retracted when functional neurotransmission recovers at the original terminal [2]. The vulnerability of intra-ganglionic parasympathetic nerve endings may be related to their dynamic plasticity, which persists well into adulthood in mouse SMG [4,5]. Considerable changes are known to occur in the position and number of presynaptic terminals of the pre-ganglionic nerves in SMG over periods ranging from days to weeks [4,5], and time-lapse microscopy has revealed dynamic structural re-arrangements (growth and retraction) over minutes [5]. Intact SNAP-25 is required for axonal growth [21], a process susceptible to BoNT/A in vitro [22,23], and though much greater amounts are required to fully retard neurite outgrowth in cultured sympathetic ganglionic neurons than are needed to block cholinergic transmission, a partial reduction in growth was observed with as little as 10 pM BoNT/A [22]. By contrast, BoNT/A inhibited vesicle docking at active zones but did not seem to reduce overall vesicle numbers in cultured spinal cord neurons [24]. Hence, it is intriguing to find that the biochemical composition of parasympathetic neurons is perturbed upon intoxication in vivo with doses similar to those needed to inhibit synaptic communication. Another possibility is that blockade of cholinergic transmission results in a decline in the production of neurotrophic factors by postsynaptic cells [25,26,27].

It is remarkable also that the paralysis of neurotransmission should be accompanied by a dramatic reduction in the expression of α7nAChRs on the surface of the postsynaptic cells. Autonomic ganglion neurotransmission is largely mediated by α3nAChRs, as evidenced by the severe dysautonomia in α3 knock-out mice [28] and the prevalence of α3-reactive antibodies in the serum from patients with autoimmune autonomic neuropathies [29]. Nevertheless, a large fraction of neurotransmission involves α7nAChRs, as revealed by a 47% blockade by α-BuTx of ganglionic transmission in mouse SMG [11] and a 21–40% block of acetylcholine-evoked currents in neurons isolated from the superior cervical ganglion of rats [30]. Furthermore, after electrical stimulation of the preganglionic nerve, the concentration of acetylcholine around α−BuTx binding sites in mouse SMG reaches levels functional for α7nAChRs excitation [31,32]. Depletion of α7nAChRs precedes the loss of presynaptic terminals in SMG after axotomy of the postganglionic fibres [33], although presynaptic blockade of neurotransmission induced loss of presynaptic SV2 before α7nAChR. Nevertheless, the overall decline and recovery of these proteins were otherwise similar. Thus, it seems that both post- and presynaptic insults cause changes in the biochemical composition on both sides of parasympathetic synapses. The overwhelmingly presynaptic localisation of the BoNT/A acceptor SV2 (Figure 3 and see [5]) is most consistent with an exclusively presynaptic block of neurotransmission, but it cannot be excluded that some BoNT/A could enter the postsynaptic ganglionic neurons and possibly inhibit delivery of α7nAChR to the cell surface [34]. Interestingly, the drastic loss of α7nAChRs observed here in intoxicated SMG differs from the persistence of muscle acetylcholine receptors in the postsynaptic membranes of BoNT/A-paralysed NMJs [2]. In this regard, it is notable that nAChRs on normal SMG neurons have been estimated to turn over ~60-fold more rapidly than those at NMJs, and they also display a ~10-fold faster rate of lateral diffusion [11]. Thus, a picture is emerging of both pre- and postsynaptic elements of parasympathetic neuro-neuronal synapses in the SMG being plastic and dynamic compared to the NMJ in adult mice. Therefore, it is reasonable to speculate that this characteristic underlies a vulnerability to changes in functional components upon blockade of synaptic transmission.

The results herein offer insight into differences in the clinical outcomes achieved when using BoNT/A (e.g., BOTOX^®^) to alleviate nerve overactivity in secretory glands as opposed to muscles, most notably the more prolonged effectiveness in ameliorating hypersecretion compared to muscle spasms (see Introduction) for some patients. The diminution of synaptic proteins in the SMG may compound the consequences of the persistence of BoNT/A protease and, thereby, contribute to delaying recovery of synaptic function. Nevertheless, it is reassuring that the synaptic proteins and activity eventually recover fully after long-term inactivation of SNAP-25. Advantageously, ganglionic blockade extends the therapeutic application of BoNT. For example, a small pilot study gained evidence that injection of BoNT/A into the lumbar sympathetic chain provided patients with some relief from the symptoms of complex regional pain syndrome [35]. However, further work is needed to define the contribution of ganglionic blockade to suppression by BoNT/A of salivation and its recovery. Other important factors to consider include the additional inhibition by BoNT/A of neurotransmission from postganglionic SMG neuron fibres at their neuroeffector junctions [16,36], the potential role of acinar cell atrophy in the denervated salivary gland [37,38], the hypersensitisation of acinar cells to acetylcholine arising from chronic low stimulation [19], and how the latter might be reconciled with reports of BoNT/A injections resulting in reduced M3 sub-type muscarinic acetylcholine receptor expression on acinar cells in rabbit SMG [37]. Moreover, the relationship of neurophysiological functional recovery to saliva production remains unknown; full recovery of mouse diaphragm contraction force can precede full recovery of neurophysiological measures by many days [7]. This article is an expanded version of a paper presented at the ‘Toxins 2026 8th International Conference: Basic Science and Clinical Aspects of Botulinum and Other Neurotoxins’ in Madrid, Spain, from 14 to 16 January [39].

## 4. Materials and Methods

Materials. Chemicals and alkaline phosphatase-conjugated secondary antibodies were from Merck (Arklow, Ireland), unless otherwise specified. Antibodies with the noted specificities were purchased: SNAP-25 (mouse monoclonal SMI-81 [Cambridge Bioscience, Cambridge, UK] and rabbit polyclonal serum [S9684, Merck]), VAMP2 and vAChT (rabbit polyclonal antibodies 104 211 and 139 103, respectively, from Synaptic Systems, Göttingen, Germany), NF-200 (N4142, rabbit polyclonal antibody from Merck), and α7 nAChR (ANC-007, Alomone Labs., Jerusalem). A monoclonal antibody recognising all three SV2 isoforms described by K. M. Buckley was obtained from the Developmental Studies Hybridoma Bank developed under the auspices of the NICHD and maintained by the University of Iowa, Department of Biology, Iowa City, IA, USA. Alkaline phosphatase-conjugated secondary antibodies were obtained from Merck. Fluorescent conjugates, α−BuTX tetramethylrhodamine-conjugate (α−BuTX-rhod.) and (α−BuTX-Alexa-647), fluor-labelled secondary antibodies (goat anti-mouse Alexa-594 and goat anti-rabbit Alexa-568), and NuPAGE acrylamide BisTris gels were purchased from Thermo Fisher Scientific, Dublin, Ireland. Mecamylamine was supplied by Tocris Bioscience, Bristol, UK. BoNT/A was recombinantly manufactured and purified in-house, as described previously [18], to a specific neurotoxicity in mice (after nicking) of 1 × 10^8^ medial lethality units (U) per mg.

Experimental Animals. A total of 110 mice were used in this study, including 32 untreated mice and 78 mice treated with different doses of BoNT/A, as reported in the figure legends. Thy1 mice [B6.g-Tg(Thy1-YFP)16Jr/J] were obtained from Jackson Laboratory Bar Harbor, ME, USA and housed in individually ventilated cages (21 ± 2 °C, humidity 36 ± 2% at 12/12 h light/dark cycle) and bred in-house. The size of experimental groups was determined by an independent biostatistician using PASS12 software (version 12.0.1) prior to ethical review and regulatory approval of the protocol. Power analysis was based on anticipated results of up to a ten-fold reduction in EPSP amplitude with BoNT/A. The experiment was set up as a 5 × 4 factorial ANOVA with 5 levels of time and 4 drug treatments (different BoNT/A doses). A sample size of 2 treatment combinations was determined for factorial ANOVA, and a value of 3 per treatment combination for the post hoc testing; therefore, the experiment was set up with three replicates and one control per treatment combination. During the study, certain treatment groups were excluded from analysis after optimum BoNT/A doses were identified. The researcher performing the experiments was aware of the group allocation.

In vivo administration of BoNT/A. Mice (12–16 weeks; either sex) were anesthetised by intraperitoneal (i.p.) injection of Ketamine (90 mg/kg) plus Xylazine (10 mg/kg), placed in a supine position, and the base of one salivary duct was exposed unilaterally [40]. Animals were assigned to groups using the random number generation function in Microsoft Excel. BoNT/A [in saline containing 0.5% bovine serum albumin (BSA); 5 μL] was injected topically with a Hamilton microsyringe into a well created around the duct using the surrounding connective tissue. In control mice, the vehicle was applied. Untreated SMGs from naïve animals were used as controls for immunostaining. Post-operative analgesia (0.2 mL of 0.25% bupivacaine hydrochloride) was administered if necessary. Mice were euthanised if they displayed any signs of respiratory distress, post-operative wound infection, or if they lost more than 20% of their body weight relative to their pre-operation measurement.

Electrophysiological measurements. Mice were euthanised with sodium pentobarbital in PBS (600 mg/kg, i.p.). Salivary ducts were dissected together with the lingual nerve attached, placed in a recording chamber [as previously detailed [13,41] and perfused with carboxygenated (95% O_2_/5% CO_2_) Krebs solution (in mM): 136 NaCl, 5 KCl, 2.5 CaCl_2_, 0.5 MgCl_2,_ 11.9 NaHCO_3_, 1.1 NaH_2_PO_4_, and 10.9 glucose (pH 7.4 with NaOH). SMGs were visualised using an Olympus BX51WI microscope by virtue of the yellow fluorescent protein (YFP) in fibres innervating the ganglia (Figure 1A) and were impaled for whole-cell recordings. This involved the use of a sharp recording pipette (borosilicate glass pulled with a Sutter Instruments Puller P-97) filled with 1 M KCl, having resistances between 50 and 65 MΩ. The lingual nerve was mounted in a suction electrode and subjected to electrical stimulation (0.5–1 ms wide pulses of 10 V) using an A365 Stimulus Isolator (WPI, Hitchin, UK) to evoke excitatory postsynaptic potentials (EPSPs) in the SMG neurons. Evoked EPSPs were recorded under current clamp from individual SMG neurons only if they exhibited resting membrane potentials more negative than −60 mV, no significant drift (±10 mV), and input resistance > 100 MΩ. Recording was performed under continuous perfusion with oxygenated external (bath) medium (in mM): 150 NaCl, 5 KCl, 10 CaCl_2_, 1 MgCl_2_, 10 Hepes, and 10 glucose (pH 7.4 with NaOH) at a rate of ~2–3 mL/min. Data were sampled at 20 kHz using an EPC10USB amplifier controlled with PatchMaster software (version 2X92; HEKA, Lambrecht, Germany) and digitised at 5 kHz for storage and off-line analysis with FitMaster software (version 2X92; HEKA Elektronik).

Western blotting. After recordings, tissues were dissolved in SDS sample buffer, heated (80 °C, 5 min), and then subjected to SDS-PAGE on NuPAGE 12% acrylamide, Bis-Tris gels. Proteins were subjected to Western blotting with rabbit polyclonal antibodies to an N-terminal region of SNAP-25, as described previously [42], or the α7 nAChR. Cleavage of SNAP-25 by BoNT/A resulted in the appearance of a faster-migrating second immuno-reactive band; estimates of the extents of SNAP-25 proteolysis were calculated by quantification of both signals using Image J version 1.48 (NIH) [42]. 

Immuno-staining of proteins, fluorescent labelling of AChRs and confocal imaging. Salivary ducts were fixed with 4% paraformaldehyde (PFA; in PBS) for 2 h, permeabilised with antibody diluent solution [AbD; 0.5% (*v*/*v*) Triton X-100 and 0.1% BSA in PBS] (24 h at 4 °C), then incubated with 5% foetal bovine serum (FBS) in AbD for 2 h at room temperature. For SV2 staining, the primary antibody was applied in FBS-AbD at 7.5 μg/mL for 2 days. Similar procedures were followed for antibodies against VAMP2 (2 μg/mL), SNAP-25 (SMI 81 ascites, 1:500), vAChT (2.5 μg/mL), and NF-200 (ascites, 1:200). After rinsing in 1% (*v*/*v*) Triton X-100 plus 5% FBS in PBS (3 × 1 h), followed by 0.1% Triton X-100 in PBS (3 × 10 min), samples were incubated overnight (4 °C) with Alexa fluor-labelled secondary antibodies (2 μg/mL) before washing with 0.1% Triton X-100 in PBS (3 × 30 min) and mounting on glass slides with Mowiol^®^ 4-88. To label nAChRs, freshly dissected salivary ducts were incubated with oxygenated PBS containing α−BuTX-rhodamine or Alexa-647-labelled α−BuTX (5 μg/mL) for 10–30 min prior to washing and fixation, incubation with antibodies, and mounting as above.

Imaging was performed with a LSM 710 AxioObserver confocal microscope (Carl Zeiss, Oberkochen, Germany). Field micrographs were obtained (20× objective) in epifluorescence mode, and high-magnification images of SMGs were obtained in confocal mode [pinhole = 1 AU, 40× objective (EC Plan-Neofluor 11.3 oil DIC M27)]. High-resolution Z-stacks were constructed from images captured using 2× digital zoom and a 63× objective (Plan-Apochromat 63×/140 oil DIC M27). Argon and helium/neon lasers provided the 514 and 543 nm lines for excitation; emitted signals were sampled in a frame mode at a spatial resolution of 30 nm per pixel with 1.3 µs dwell time.

Data analysis. Intensities of fluorescence were measured for equal-sized regions of interest (ROIs) on single optical sections, using Zen 2008 software (Carl Zeiss, Germany). The mean pixel value in each region was scored, and the background reading was subtracted. Confocal planes above and below the ROI were similarly analysed, and the data reported as mean ± s.e.m.; *n* values refer to the number of individual neurons examined. Electrophysiological data were obtained by averaging the EPSP amplitudes acquired in each experiment; *n* = 4–9 neurons were recorded from accessible ganglia taken from each group of mice (2–5 animals) subjected to 0.1 or 0.5 U doses of BoNT/A. The percentage of inhibition was presented as the mean ± the standard error of the mean.

Statistical significance was evaluated by an unpaired two-tailed Student’s *t*-test, using data obtained from at least 3 independent experiments on 2 to 5 animals. *p*-values < 0.05 were considered significant, with the following levels of statistical significance: * indicates a *p* < 0.05; ** indicates a *p* < 0.01; and *** *p* < 0.001.

Study limitations. Small numbers of replicates were utilised for Western blots and immunohistochemistry because group sizes were determined for electrophysiological recordings only. The concentrations of BoNT/A used were limited by respiratory effects at higher doses, and the relationship of the doses used here to those used clinically in humans has not been established.

## Data Availability

The original contributions presented in this study are included in the article/Supplementary Material. Further inquiries can be directed to the corresponding authors.

## Supplementary Materials

The following supporting information can be downloaded at: https://www.mdpi.com/article/10.3390/toxins18010043/s1, Figure S1: Cleaved SNAP-25 is detected in mouse salivary ducts within days of exposure to different doses of BoNT/A; Figure S2: Western blotting detects the presence of the a7 nAChR in the mouse salivary duct; Figure S3: SV2 and nAChR levels of expression are unchanged in untreated SMGs and those pre-treated with inactive BoNT/A (BoTIM/A).

## Abbreviations

The following abbreviations are used in this manuscript:

α-BuTX	α-bungarotoxin
BoNT/A	botulinum neurotoxin type A
EPSPs	excitatory postsynaptic potentials
NF-200	neurofilament-200
nAChR	nicotinic acetylcholine receptor
PBS	phosphate-buffered saline
PFA	para-formaldehyde
SNAP-25	synaptosomal-associated protein of Mr = 25
SNARE	soluble N-ethyl-maleimide-sensitive factor attachment protein receptor
SV2	synaptic vesicle protein 2
VAMP	vesicle-associated membrane protein
vAChT	vesicular acetylcholine transporter
YFP	yellow fluorescent protein
