# Supplementary material for: Presynaptic Terminal Proteins and Nicotinic Receptors Are Depleted from Mouse Parasympathetic Ganglionic Junctions Paralysed with Botulinum Neurotoxin Type A
Source: Toxins (Basel). 2026 Jan 14;18(1):43. doi: 10.3390/toxins18010043 (PMC12846174; doi:10.3390/toxins18010043)
Supplement: Supplementary file 1 [file toxins-18-00043-s001.zip › toxins-4080174-supplementary.pdf]

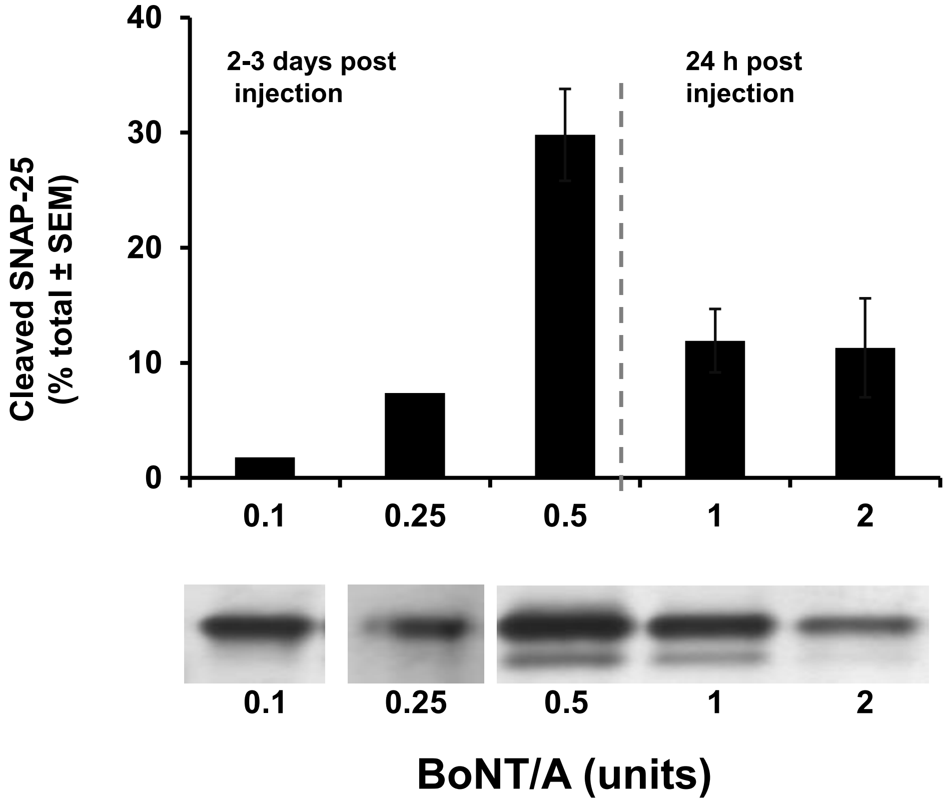

Figure S1: Cleaved SNAP-25 is detected in mouse salivary ducts within days of exposure to different doses of BoNT/A. SNAP-25 cleavage 2-3 days after injection increased dose-dependently up to 0.5 U BoNT/A. Animals exposed to 1 or 2 U of BoNT/A were sacrificed after 24 due to the appearance of systemic symptoms, with cleavage of SNAP-25 already evident. The plot displays data quantified from Western blots obtained for single animals (0.1 and 0.25 U) and mean values from two (2 U), three (1 U) or five animals (0.5 U).

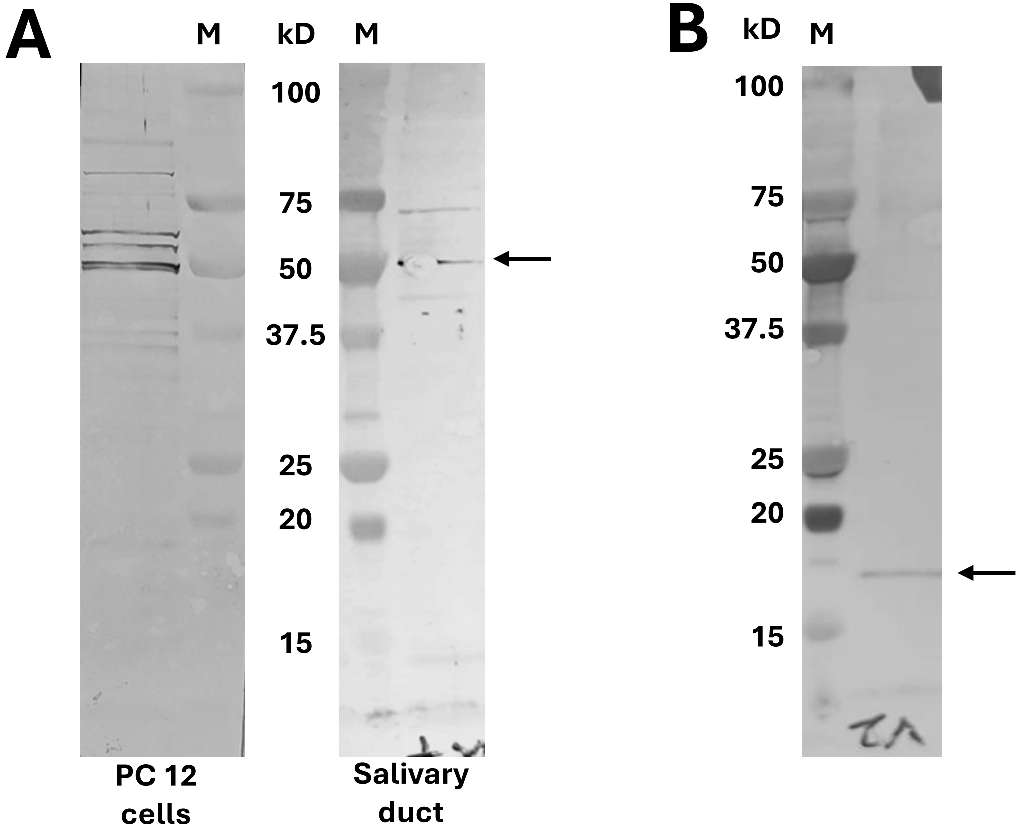

Figure S2: Western blotting detects the presence of the α7 nAChR and VAMP2 in the mouse salivary duct
Detergent-solubilised extracts of rat PC 12 cells and mouse salivary duct were subjected to SDS-PAGE followed by protein transfer to PVDF and Western blotting with an antibody to (**A**) the α7 nAChR or (**B**) VAMP2. In (**A**) the major band just above the 50 kD molecular weight marker (in lanes labelled M) corresponds to the expected size of a single α7 nAChR subunit (Mr 54 kD). The bands of higher molecular weight are likely to be post-translationally modified α7 nAChR sub-units. In (**B**) the antibody selectively bound to a protein of Mr 18 k, the expected size for VAMP2.

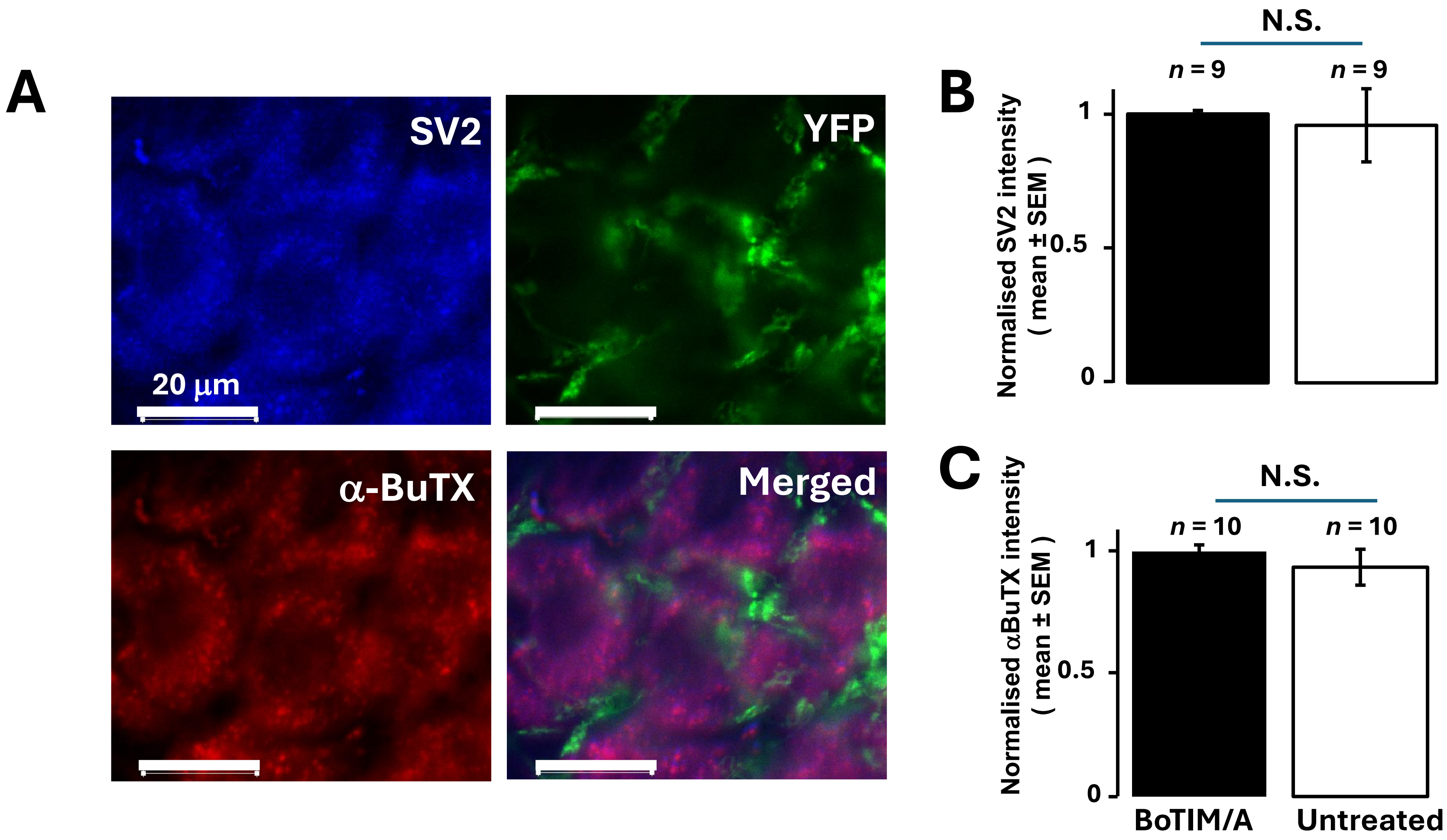

Figure S3: SV2 and nAChR levels of expression are unchanged in untreated SMGs and those pre-treated with inactive BoNT/A (BoTIM/A) (A) Confocal 3D micrographs of SMG neurons showing fluorescent signals of pre-synaptic SV2 (Upper left panel) and post-synaptic α7 nAChR visualised by α-BuTX (Lower left panel). Both proteins are expressed in SMGs of mice pre-injected 7 days previously with 10 pg BoTIM/A, which is equivalent to the mass of 1U of BoNT/A. **(B)** and **(C)** Relative fluorescence intensities for α-BuTX binding sites (α7 nAChRs) and indirect immunodetection of SV2; no significant (N.S) difference between BoTIM/A and untreated SMGs was detected for either fluorescent signal.
